# Dietary Nitrate Metabolism in Porcine Ocular Tissues Determined Using ^15^N-Labeled Sodium Nitrate Supplementation

**DOI:** 10.3390/nu16081154

**Published:** 2024-04-13

**Authors:** Ji Won Park, Barbora Piknova, Khalid J. Tunau-Spencer, Samantha M. Thomas, Hongyi Cai, Peter J. Walter, Audrey Jenkins, David Hellinga, Leonard M. Parver, Alan N. Schechter

**Affiliations:** 1 Molecular Medicine Branch, National Institute of Diabetes and Digestive and Kidney Diseases, Bethesda, MD 20892, USA; jiwon.park@nih.gov (J.W.P.); piknovab@mail.nih.gov (B.P.); khalid.j.tunau.spencer@gmail.com (K.J.T.-S.); samanthathomas188@gmail.com (S.M.T.); 2 Molecular Medicine Branch, National Institutes of Health, Bethesda, MD 20892, USA; 3 Clinical Mass Spectrometry Core, National Institute of Diabetes and Digestive and Kidney Diseases, Bethesda, MD 20892, USA; hongyi.cai@nih.gov (H.C.); walterpj@niddk.nih.gov (P.J.W.); 4 Clinical Mass Spectrometry Core, National Institutes of Health, Bethesda, MD 20892, USA; 5 MedStar Health Research Institute, Washington, DC 20010, USA; audrey.jenkins@medstar.net (A.J.); david.g.hellinga@medstar.net (D.H.); 6 Department of Ophthalmology, Medstar Georgetown University Hospital, Washington, DC 20007, USA

**Keywords:** dietary nitrate, nitrite, nitric oxide, porcine eye

## Abstract

Nitrate (NO_3_^−^) obtained from the diet is converted to nitrite (NO_2_^−^) and subsequently to nitric oxide (NO) within the body. Previously, we showed that porcine eye components contain substantial amounts of nitrate and nitrite that are similar to those in blood. Notably, cornea and sclera exhibited the capability to reduce nitrate to nitrite. To gain deeper insights into nitrate metabolism in porcine eyes, our current study involved feeding pigs either NaCl or Na^15^NO_3_ and assessing the levels of total and ^15^N-labeled NO_3_^−^/NO_2_^−^ in various ocular tissues. Three hours after Na^15^NO_3_ ingestion, a marked increase in ^15^NO_3_^−^ and ^15^NO_2_^−^ was observed in all parts of the eye; in particular, the aqueous and vitreous humor showed a high ^15^NO_3_^−^ enrichment (77.5 and 74.5%, respectively), similar to that of plasma (77.1%) and showed an even higher ^15^NO_2_^−^ enrichment (39.9 and 35.3%, respectively) than that of plasma (19.8%). The total amounts of NO_3_^−^ and NO_2_^−^ exhibited patterns consistent with those observed in ^15^N analysis. Next, to investigate whether nitrate or nitrite accumulate proportionally after multiple nitrate treatments, we measured nitrate and nitrite contents after supplementing pigs with Na^15^NO_3_ for five consecutive days. In both ^15^N-labeled and total nitrate and nitrite analysis, we did not observe further accumulation of these ions after multiple treatments, compared to a single treatment. These findings suggest that dietary nitrate supplementation exerts a significant influence on nitrate and nitrite levels and potentially NO levels in the eye and opens up the possibility for the therapeutic use of dietary nitrate/nitrite to enhance or restore NO levels in ocular tissues.

## 1. Introduction

Dietary nitrate, abundant in green leafy vegetables and beetroots, has been demonstrated to enhance NO bioavailability through two-step reduction mechanisms [1,2,3]. Once it is ingested, nitrate can be reduced to nitrite by oral commensal bacteria [4,5] and, to some extent, by mammalian molybdenum (Mo)-containing enzymes such as xanthine oxidoreductase (XOR) [6,7,8]. Nitrite ions can then be further reduced to NO via several mechanisms [9,10,11,12]. Numerous studies demonstrated the beneficial effects of dietary nitrate consumption on the cardiovascular system, such as lowering blood pressure and increasing blood flow [13,14,15], as well as improving exercise efficiencies [16,17,18]. Interestingly, recent epidemiological studies have shown that the intake of dietary nitrate was inversely associated with the development of several eye diseases, such as age-related macular degeneration and glaucoma [19,20,21,22,23]. Also, some eye diseases, namely myopia and glaucoma, are known to be linked to a reduction in NO bioavailability [24,25].

NO has been considered a crucial regulator for intraocular pressure, due to its roles in enhancing aqueous humor outflow via the relaxation of trabecular meshwork [26,27]. The importance of NO signaling in the eye via nitric oxide synthase (NOS) pathways for the regulation of ocular blood flow has been recognized [28,29], since all three NOS isoforms have been identified in mammalian ocular tissues [30]. Recently, the FDA approved an NO-donating prostaglandin analogue, latanoprostene bunod, for reducing intraocular pressure in patients with an open-angle glaucoma or ocular hypertension [31]. However, the contribution of the nitrate–nitrite–NO reduction pathways to NO bioavailability and involvement in physiological and pathophysiological phenomena in the eye has not been greatly appreciated so far. Given that the nitrate–nitrite–NO pathways are considered significant sources of bioavailable NO in various organ systems, it is now thought that NO produced from nitrate reduction pathways could play a role in the ocular system. In our previous report, we showed that several ocular components in pigs contain substantial amounts of nitrate and nitrite ions [32]. More importantly, the cornea and sclera exhibited a nitrate reduction activity, suggesting that dynamic nitrate–nitrite–NO pathways may exist and take part in ocular signaling pathways. However, questions remain on the extent of the contribution of each NO source, namely NOS and nitrate reduction pathways, to the overall NO metabolism in the eye. One of the quantitative methods used to calculate the contribution of each NO source is to use stable isotope-labeled supplements and monitor the amount of tracers accumulated in target tissues. Previously, we and others have used ^15^N-labeled nitrate, nitrite, or arginine to determine the incorporation of these supplements in plasma or other tissues such as skeletal muscle [33,34,35,36]. Specifically, in a rat study, we showed that the eyes took in dietary nitrate more efficiently than other major organs (liver or skeletal muscle); also, the amount of nitrite generation from the exogenous nitrate was higher in the eyes compared to that in the liver and skeletal muscle [37], which suggests that the eye is an active organ that can metabolize dietary nitrate. In the present study, we examined the nitrate metabolism in the eye in more detail by obtaining different parts of porcine ocular tissues after ^15^N-labeled nitrate administration.

## 2. Materials and Methods

### 2.1. Animal Study

This research was conducted as part of the animal protocols approved by the IACUC (Institutional Animal Care and Use Committee, protocol # 2020-017) of Medstar Health Research Institute, in compliance with the Animal Welfare Act and the Guide for the Care and Use of Laboratory Animals, 8th ed. Both male and female Yorkshire domestic cross swine, weighing between 35 and 40 kg, sourced from Thomas D. Morris, Inc. (Reisterstown, MD, USA) were used. On arrival, animals were acclimated for a minimum of 72 h and housed in an AAALAC (Association for Assessment and Accreditation of Laboratory Animal Care)-accredited facility with environmental enrichment. Animals were fed twice daily with a commercial chow (Teklad miniswine diet, 8753C, Envigo, Madison, WI, USA). Fresh water was provided ad libitum via an automated system. Na^15^NO_3_ (0.15 mmol/kg) or NaCl (0.15 mmol/kg) were given orally with a moist food ball of swine chow.

On the day of terminal tissue collection, pigs were sedated with a cocktail mixture of ketamine (15–20 mg/kg, Zoetis, Parsippany, NJ, USA) and xylazine (3–5 mg/kg, Covetrus, Dublin, OH, USA) and were then maintained under 3–5% isoflurane with 2 L/min oxygen anesthesia, with a mechanical ventilator, during the entire procedure. Both the left and right femoral veins were cannulated via ultrasound-guided percutaneous access; the left or right common carotid artery and jugular vein were accessed using a 2–3-inch ventral midline neck skin incision. Tygon 3350 silicone tubing (Saint-Gobain, Williamsburg, MI, USA) was connected to all venous sheaths and animals were heparinized (≥200 IU/Kg). Blood removal began 15 min after heparin administration with all three venous access lines. Euthanasia was accomplished with a single intravenous injection of saturated potassium chloride (4.6 mL per 10 kg body weight, IV bolus, using a 4.2 M KCl concentration), while the animals were maintained on isoflurane gas anesthesia, as per the AVMA (American Veterinary Medical Association, Schaumburg, IL, USA) guidelines. Perfusion began immediately after euthanasia, with the arterial sheath attached to warm 0.9% NaCl solution (Baxter Healthcare, Mississauga, ON, Canada) with heparin (2000 IU/L, Fresenius Kabi, Melrose Park, IL, USA). A total of 8 L of heparinized 0.9% NaCl solution was perfused before ocular tissue collection.

### 2.2. Sample Preparation for Nitrate and Nitrite Measurements

Standard chemiluminescence assays for measuring nitrite and nitrate contents were performed according to previously published protocols [38,39]. Blood was drawn from the femoral artery into vacutainer tubes containing sodium citrate (Becton Dickinson, Franklin Lakes, NJ, USA) and was immediately centrifuged to obtain platelet-free plasma using a PDG_TM_ platelet centrifuge (Bio/Data, Horsham, PA, USA). Tissue samples were collected and proteins from all samples were precipitated by adding methanol (dilution 1:1), followed by subsequent centrifugation at 11,000× *g* for 15 min at 4 °C. Supernatants were used to determine nitrite and nitrate contents using chemiluminescence (Sievers 280i Nitric Oxide Analyzer, GE Analytical Instruments, Boulder, CO, USA).

### 2.3. Preparation of Samples for Liquid Chromatography–Tandem Mass Spectrometry (LC–MS/MS)

To measure nitrate content using LC-MS/MS, nitrate ions in all samples were first enzymatically reduced to nitrite by bacterial nitrate reductase from *Aspergillus niger* (N7265, Sigma-Aldrich, St. Louis, MO, USA), as previously described [40,41], with some modification. Briefly, the sample (20 µL) was mixed with 96 µL of a mixture consisting of nitrate reductase (0.1 U/mL) and nicotinamide adenine dinucleotide phosphate (NADPH, 100 µM) and was incubated for 2 h at room temperature. Then, nitrite ions in the samples were derivatized with 2,3-diaminonaphthalene (DAN, D2757, Sigma-Aldrich) for 30 min at 37 °C to yield 2,3-naphthotriazole (NAT). NaOH (58 mM) was added to terminate the reaction. For measuring nitrite content only, the samples (50 µL) were directly subjected to DAN derivatization.

### 2.4. Determination of ^15^NO_3_^−^ or ^15^NO_2_^−^ Percent Using LC–MS/MS

High-performance liquid chromatography (HPLC)-grade solvents and LC–MS modifiers were purchased from Sigma-Aldrich (St. Louis, MO, USA). Detection and quantification were achieved using ultra-performance liquid chromatography–tandem mass spectrometry (UPLC–MS/MS), utilizing a Thermo Scientific Vanquish UPLC (Thermo Fisher Scientific, Waltham, MA, USA) with a Thermo Scientific Altis triple quadrupole mass spectrometer, with a heated electrospray ionization (HESI-II) in positive ion mode (3500 V). In total, 50 µL of sample was mixed with 200 µL of acetonitrile (ACN), vortexed for 5 min, and was then centrifuged at 4 °C at 17,000× *g* for 15 min. The supernatant was transferred to an LC–MS vial for analysis. The injection volume was 1 µL. A Waters Cortecs T3, 2.1 × 100 mm, 1.6 µm column was maintained at 35 °C. Solvent A consisted of H_2_O with 0.1% formic acid (FA) and Solvent B consisted of ACN with 0.1% FA. The flow rate was 250 µL/min, the gradient was 25% B at 0 min for 0.25 min, increasing to 65% B at 5 min, further increasing to 90% B at 5.5 min, remained at 90% B until 7.5 min, and then decreased to 25% B at 8 min. The total running time was 10 min. Samples were analyzed in triplicates. Quantitation of ^14^NAT and ^15^NAT were based on multiple reaction monitoring (MRM) transitions *m*/*z*, 170.062 → 115.042 and 171.062 → 115.042, respectively. The result was based on the percentage ratio of ^15^NAT/(^14^NAT + ^15^NAT).

### 2.5. Statistical Analysis

Values represent average ± standard deviation. The statistical significance of the results was tested using a one-way ANOVA. * denotes *p* < 0.05.

## 3. Results

To assess the effect of dietary nitrate supplementation on the amounts of nitrate and nitrite ions incorporated in different parts of the eye, we orally administered either placebo (NaCl) or Na^15^NO_3_ (0.15 mmol/kg) to Yorkshire domestic pigs (35–40 kg), then collected and dissected each eye into several different components at either the 3 h or 24 h time point, following administration. The cornea, sclera, lens, retina, optic nerve, and aqueous and vitreous humor were harvested. In addition, the lacrimal gland, ocular muscle, and plasma were collected for comparison.

**Figure 1:**
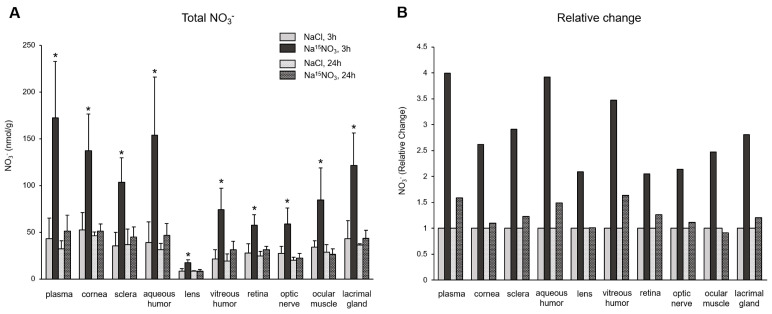
Total nitrate concentration (panel **A**) and its relative change (panel **B**) in porcine plasma and ocular tissues after Na^15^NO_3_ administration. (**A**) Tissue samples were homogenized using a bead homogenizer, then centrifuged (17,000× *g*, 30 min) after mixing with methanol for deproteinization [39]. The supernatant was used for nitrate measurement using a standard chemiluminescence method with vanadium chloride [38]. Data are plotted as average ± standard deviation (*n* = 4 for 3 h groups, *n* = 3 for 24 h groups, * *p* < 0.05 compared to NaCl 3 h). N represents an individual animal. (**B**) The change in each tissue at 3 h and 24 h after Na^15^NO_3_ administration relative to NaCl administration.

Figure 1 shows the total concentrations of nitrate (Figure 1A) and its relative changes (Figure 1B) in ocular tissues after the nitrate administration. Nitrate levels in all ocular tissues and plasma were significantly higher when supplemented with Na^15^NO_3_, compared to NaCl at 3 h; plasma exhibited the highest fold change among all the samples (a 4-fold increase), but aqueous and vitreous humor showed the highest fold changes in ocular tissues (3.9- and 3.5-fold increase, respectively), followed by the sclera, lacrimal gland, and cornea (2.9-, 2.8-, and 2.6-fold increase, respectively). The values of nitrate at 24 h after Na^15^NO_3_ supplementation were similar or slightly higher than the control NaCl, but considerably lower than those observed at the 3 h time point.

**Figure 2:**
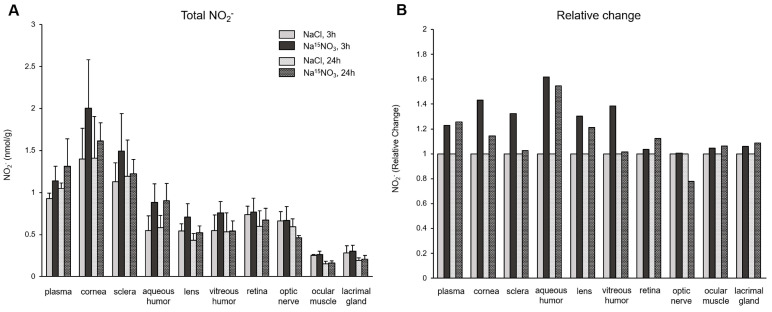
Total nitrite concentration (panel **A**) and its relative change (panel **B**) in porcine plasma and ocular tissues after Na^15^NO_3_ administration. (**A**) Tissue samples were homogenized using a bead homogenizer, then centrifuged (17,000× *g*, 30 min) after mixing with methanol for deproteinization [39]. The supernatant was used for nitrate measurement using a standard chemiluminescence method with tri-iodide [38]. Data are plotted as average ± standard deviation (*n* = 4 for 3 h groups, *n* = 3 for 24 h groups). N represents an individual animal. (**B**) The change in each tissue at 3 h and 24 h after Na^15^NO_3_ administration relative to NaCl administration.

In Figure 2, we analyzed the concentrations of nitrite (Figure 2A) and its relative changes (Figure 2B) in ocular tissues. The administration of Na^15^NO_3_ caused a substantial rise in nitrite concentration at 3 h in the cornea, sclera, and aqueous and vitreous humor, compared to NaCl administration. However, there were no significant changes in the retina, optic nerve, ocular muscle, and lacrimal gland, in terms of nitrite levels. The fold changes also show similar results in these ocular tissues (1.4-, 1.3-, 1.6-, and 1.4-fold increase in the cornea, sclera, and aqueous and vitreous humor, respectively, and minimal alterations were observed in other ocular tissues).

**Figure 3:**
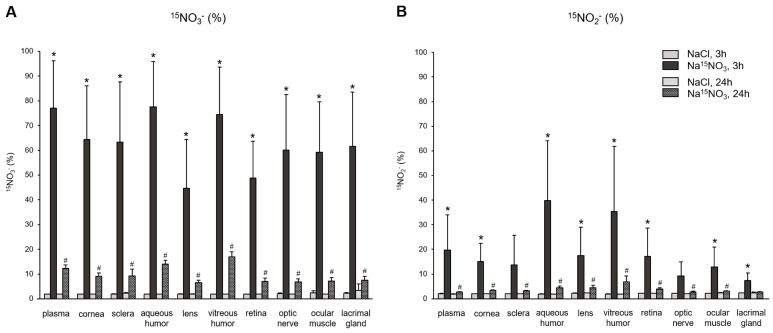
^15^N-labeled nitrate (panel **A**) and nitrite (panel **B**) content in porcine plasma and ocular tissues after Na^15^NO_3_ administration. Porcine eyes were dissected into cornea, sclera, aqueous humor, lens, vitreous humor, retina, and optic nerve. Tissue samples including the ocular muscle (superior and medial rectus) and lacrimal gland were homogenized using a bead homogenizer [39], then centrifuged (17,000× *g*, 30 min). ^15^N-labeled nitrate and nitrite contents were measured using LC–MS/MS. All nitrate ions in samples were first reduced to nitrite by bacterial nitrate reductases (*Aspergillus niger*). Then, nitrite derivatization using DAN was performed to yield NAT. The result was based on the percentage ratio of ^15^NAT/(^14^NAT + ^15^NAT). Data are plotted as average ± standard deviation (*n* = 4 for 3 h groups, *n* = 3 for 24 h groups). N represents an individual animal. * *p* < 0.05 compared to NaCl 3 h, ^#^ *p* < 0.05 compared to NaCl 24 h.

Next, to provide a precise assessment of the incorporation of dietary nitrate (supplemented Na^15^NO_3_) and its metabolite, nitrite, into ocular tissues, we employed LC–MS/MS, as described in the Section 2 (Materials and Methods Section), to calculate the percent of ^15^N-labeled nitrate and nitrite in the tissue samples. The baseline level of ^15^N-labeled nitrate or nitrite obtained from NaCl-supplemented pigs was approximately 2% in all samples. Figure 3A shows the amount of ^15^NO_3_^−^, based on the ratio of ^15^N/(^15^N + ^14^N), in plasma and various ocular tissues. After 3 h of nitrate administration, the percent of ^15^NO_3_^−^ in all samples was significantly higher than that in NaCl-administered samples. Among the ocular tissues, the aqueous and vitreous humor showed the highest incorporation of exogenous nitrate (77.5% and 74.5%, respectively), which was similar to that of plasma (77.1%). However, 24 h after nitrate administration, the percentage of ^15^NO_3_^−^ in all samples decreased markedly, but still remained higher than the baseline observed in NaCl-administered pig samples. In Figure 3B, we analyzed the percentage of ^15^NO_2_^−^, which directly represents the reduction amount derived from the supplemented Na^15^NO_3_ in plasma and ocular tissues. In plasma, 3 h after the Na^15^NO_3_ administration, 19.8% of nitrite was ^15^N-labeled. Interestingly, aqueous and vitreous humor had even higher levels of ^15^NO_2_^−^ compared to plasma (39.9% and 35.3%, respectively), although these values were not statistically different from plasma. All other ocular tissues had slightly lower levels of ^15^NO_2_^−^ than plasma, but all exhibited significantly higher levels of ^15^NO_2_^−^ 3 h after the Na^15^NO_3_ administration, compared to NaCl.

**Figure 4:**
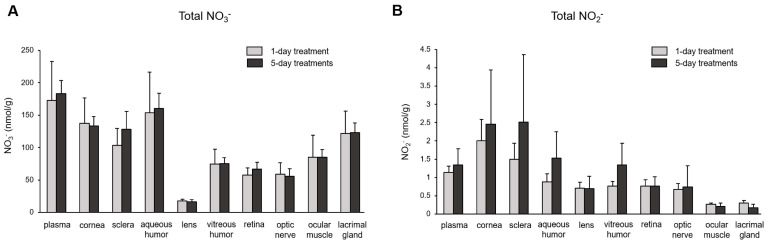
Total nitrate and nitrite concentration in porcine plasma and ocular tissues after supplementing Na^15^NO_3_ for five consecutive days. (**A**,**B**) Ocular tissues and plasma were collected from 1-day- and 5-day-treated animals and were prepared for nitrate and nitrite analysis with a standard chemiluminescence method [38]. Data are plotted as average ± standard deviation (*n* = 4). N represents an individual animal.

**Figure 5:**
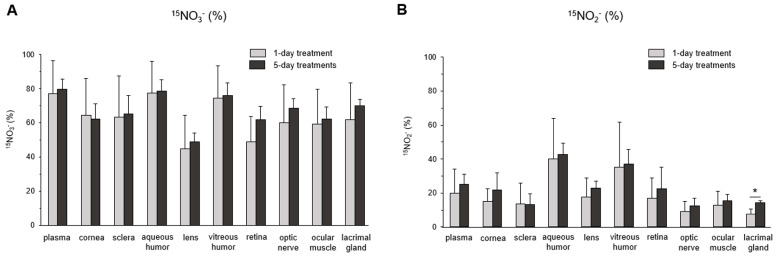
^15^N-labeled nitrate and nitrite content in porcine plasma and ocular tissues after supplementing Na^15^NO_3_ for five consecutive days. (**A**,**B**) Ocular tissues and plasma were collected from 1-day- and 5-day-treated animals and homogenates were prepared for LC–MS/MS [39]. Nitrate ions in samples were first reduced to nitrite by bacterial nitrate reductases (*Aspergillus niger*). Then, nitrite derivatization using DAN was performed to yield NAT. The result was based on the percentage ratio of ^15^NAT/(^14^NAT + ^15^NAT). Data are plotted as average ± standard deviation (*n* = 4). N represents an individual animal. * *p* < 0.05 compared to 1-day treatment.

The subsequent question we investigated was the extent of nitrate/nitrite accumulation following Na^15^NO_3_ administration for five consecutive days, in comparison to a single administration. In Figure 4, we compared the total concentration of nitrate (Figure 4A) and nitrite (Figure 4B) in plasma and ocular tissues between single and multiple treatments. However, we did not observe any significant differences in any of the ocular tissues or plasma between single and multiple treatments, although there was a slight upward trend in nitrite levels noted in the cornea, sclera, and aqueous and vitreous humor in 5-day treatments compared to a single treatment. Furthermore, our analysis of ^15^NO_3_^−^ and ^15^NO_2_^−^ did not reveal any significant differences between single and multiple nitrate treatments (Figure 5).

## 4. Discussion

Recent epidemiological studies have suggested a potential link between dietary nitrate intake and the reduced prevalence of ocular diseases such as glaucoma and age-related macular degeneration [19,20,21,22,23]. However, there is limited information available regarding how nitrate metabolism might influence various physiological pathways in the eye, through the nitrate–nitrite–NO reductive pathways. Although the earlier studies showed that NO contributes to regulating ocular blood flow and intraocular pressure [42,43,44], the majority of studies considered the NOS-mediated NO pathways as the sole source of NO.

In the present study, we aimed to investigate the contribution of the nitrate–nitrite–NO pathways in the porcine eye by examining the levels of nitrate and nitrite ions in various ocular components and surrounding tissues, such as the ocular muscle and lacrimal gland, following supplementation with either sodium chloride (placebo) or sodium nitrate. Since NO can be generated endogenously by different NOS isoforms in ocular tissues and can be, subsequently, oxidized to nitrite and nitrate, it became essential for us to establish an efficient method to distinguish between diet-derived nitrate/nitrite and NOS-derived nitrate/nitrite ions, to gain a better understanding of how dietary nitrate contributes to the NO pathways.

To determine the amount of diet-derived nitrate and nitrite, we employed an isotope-labeled nitrate (Na^15^NO_3_). The monitoring of dynamic changes in the levels of ^15^NO_3_^−^ and ^15^NO_2_^−^ in ocular tissues following Na^15^NO_3_ administration provided clear evidence that significant elevations of endogenous nitrate and nitrite concentrations in the eye can, indeed, be achieved through dietary nitrate supplementation. All ocular tissues collected 3 h after Na^15^NO_3_ administration exhibited a significant increase in nitrate concentration (Figure 1A). When we calculated the relative changes compared to NaCl supplementation (Figure 1B), we found that nitrate treatment led to a 4.0-fold increase in plasma and, to our surprise, comparable increases in nitrate levels were observed in the aqueous and vitreous humor (3.9- and 3.5-fold increases, respectively). Additionally, all other ocular tissues also showed significant increases in nitrate concentration, with the sclera and cornea showing a relatively higher uptake compared to other parts such as the lens, retina, and optic nerve. These results indicate that ocular tissues actively transport exogenously administered nitrate into the eye and this can be partially attributed to the widespread expression of the nitrate transporter, sialin, in porcine ocular tissues [32]. In addition, we, and others, have previously confirmed that the chloride channel (CLC) family can play a role in transporting nitrate ions [45,46] and ocular tissues expressing CLC [47]. However, further investigation is needed to understand the precise mechanisms by which these proteins facilitate nitrate transport in the eye. We also noted that the lacrimal glands, responsible for tear production, showed a substantial incorporation of dietary nitrate (2.8-fold) and this suggests tears may play a significant role in efficiently distributing dietary nitrate onto the ocular surface.

Consistent with the findings regarding total nitrate levels, the analysis of ^15^NO_3_^−^ content using LC–MS/MS 3 h after supplementation revealed marked increases in both ocular and plasma samples (Figure 3A). Notably, both the aqueous and vitreous humor exhibited the highest percent of ^15^NO_3_^−^ (77.5 and 74.5%, respectively), which are very close to that of plasma (77.1%). Samples from the cornea, sclera, optic nerve, ocular muscle, and lacrimal gland contained similar amounts of ^15^NO_3_^−^, approximately ranging from 59 to 64%. The retina (48.8%) and lens (44.8%) exhibited the lowest incorporation of ^15^NO_3_^−^ among ocular tissues. Our current results do not allow us to precisely determine how much ^15^NO_3_^−^ was taken up directly through tears and entered the cornea and other ocular tissues, and it is not possible for us to estimate how much ^15^NO_3_^−^ came from the circulation into these ocular tissues. However, it is clear that the eye is an active organ, capable of absorbing nitrate ions from the diet. In our previous study with rats, we compared the ^15^NO_3_^−^ incorporation in skeletal muscle, liver, and eye after Na^15^NO_3_ administration in drinking water for 3 days [37]. Surprisingly, the eye showed much higher ^15^NO_3_^−^ contents (44.3%) compared to skeletal muscle (16.9%) and liver (10.9%), which suggests that eye tissues effectively absorb and utilize dietary nitrate and could benefit from nitrate therapy in situations where there is a need to increase NO levels in pathological conditions.

In contrast to nitrate, our analysis of total nitrite concentrations in ocular tissues did not reveal any statistically significant changes, although there was a tendency towards increases in all samples, particularly in the cornea and sclera, 3 h after the nitrate administration (Figure 2). However, ^15^NO_2_^−^ analysis showed marked increases in plasma and all ocular tissues following nitrate supplementation (Figure 3B). This finding suggests that nitrate ions derived from diets and endogenous NOS systems actively participate in dynamic metabolic pathways, where they are converted into nitrite within tissues and are subsequently re-distributed. Since nitrate and nitrite ions travel from/to the circulation via either concentration-dependent diffusion or active transport facilitated by membrane transporters, the levels of nitrate and nitrite within tissues can vary based on physiological needs. Our results indicate that total nitrite concentrations may not fully represent the amount of nitrite generated from exogenously added nitrate. This implies the existence of a delicate balance within cells to maintain these anions in optimal ranges, thereby supporting NO homeostasis. However, we currently lack precise knowledge regarding how cells determine the utilization of newly introduced dietary nitrate versus the existing nitrate pool produced by NO oxidation. Consuming the existing (“in-cell”) nitrate/nitrite first and then replenishing the endogenous pool of these anions through supplementation may be necessary to preserve stable NO signaling. Further research is needed to elucidate the intricate mechanisms underlying these processes.

Upon ingestion, a portion of dietary nitrate becomes involved in metabolic pathways, while the remainder is excreted in urine [48]. Previous studies have shown that elevated nitrate levels in plasma and skeletal muscle return to the baseline 24 h after a bolus administration [49]. Our present results also demonstrate that total nitrate levels were not statistically different between the NaCl and Na^15^NO_3_ groups 24 h after administration (Figure 1). However, we noted that the ^15^NO_3_^−^ and ^15^NO_2_^−^ contents in ocular tissues remained statistically higher in the Na^15^NO_3_ group compared to the NaCl group at the 24 h mark (Figure 3A,B). This led us to investigate whether repetitive nitrate administration for five consecutive days could result in even higher levels of both total and ^15^N-labeled nitrate/nitrite compared to a bolus administration (Figure 4 and Figure 5). However, our results revealed no notable differences in nitrate/nitrite levels between the 1-day and 5-day treatment groups in either plasma or ocular tissues. These results suggest that there is no imperative need to supply dietary nitrate for an extended duration to achieve supraphysiological levels of nitrate and nitrite, at least in the eye. Nevertheless, it may be important to supply dietary nitrate daily to ensure a persistent source of NO when required.

Dietary nitrate consumption can be a very efficient means of achieving elevated NO levels within the body. Once absorbed from the diet, nitrate can travel and be re-distributed to various tissues in the form of nitrate itself or as nitrite after conversion, while NO is rapidly scavenged within the blood and tissues [50,51]. In a previous study, we demonstrated the existence of reductive nitrate pathways in the porcine eye, highlighting the capability of the cornea and sclera tissues to convert nitrate to nitrite [32]. Importantly, the environment within the eye is known to be relatively hypoxic [52] and the aqueous humor contains approximately 20 times higher concentrations of ascorbic acid than plasma [53,54], which contributes to creating favorable conditions for nitrite reduction to NO [9] in the eye. Collectively, these studies, along with our current results, suggest that nitrate reductive pathways may serve as a crucial mechanism for maintaining NO homeostasis in the eye. Further investigations at the cellular level are needed to uncover the precise regulatory mechanisms through which ocular cells effectively utilize dietary nitrate. Understanding these mechanisms can provide valuable insights into the role of dietary nitrate in ocular health and may lead to potential therapeutic applications.

## 5. Conclusions

In the present study, we supplemented pigs with ^15^N-labeled sodium nitrate to evaluate the influence of exogenous nitrate on the levels of NO metabolites in the eye. We demonstrated that dietary nitrate administration directly raises the endogenous levels of nitrate and nitrite in ocular tissues, as well as in circulation, using LC–MS/MS. Given the critical roles of NO in enhancing the aqueous humor outflow and subsequently reducing intraocular pressure, boosting the nitrate–nitrite–NO reductive pathways to elevate NO bioavailability in the eye could be a safe and effective approach to improving ocular health.

## Data Availability

The raw data supporting the conclusions of this article will be made available by the corresponding author upon reasonable request due to in agreement with the NIH practice.
